# Low doses of oxygen ion irradiation cause long-term damage to bone marrow hematopoietic progenitor and stem cells in mice

**DOI:** 10.1371/journal.pone.0189466

**Published:** 2017-12-12

**Authors:** Yingying Wang, Jianhui Chang, Xin Li, Rupak Pathak, Vijayalakshmi Sridharan, Tamako Jones, Xiao Wen Mao, Gregory Nelson, Marjan Boerma, Martin Hauer-Jensen, Daohong Zhou, Lijian Shao

**Affiliations:** 1 Division of Radiation Health, Department of Pharmaceutical Sciences, University of Arkansas for Medical Sciences, Little Rock, AR, United States of America; 2 Department of Basic Sciences, Division of Radiation Research, School of Medicine, Loma Linda University, Loma Linda, CA, United States of America; ENEA Centro Ricerche Casaccia, ITALY

## Abstract

During deep space missions, astronauts will be exposed to low doses of charged particle irradiation. The long-term health effects of these exposures are largely unknown. We previously showed that low doses of oxygen ion (^16^O) irradiation induced acute damage to the hematopoietic system, including hematopoietic progenitor and stem cells in a mouse model. However, the chronic effects of low dose ^16^O irradiation remain undefined. In the current study, we investigated the long-term effects of low dose ^16^O irradiation on the mouse hematopoietic system. Male C57BL/6J mice were exposed to 0.05 Gy, 0.1 Gy, 0.25 Gy and 1.0 Gy whole body ^16^O (600 MeV/n) irradiation. The effects of ^16^O irradiation on bone marrow (BM) hematopoietic progenitor cells (HPCs) and hematopoietic stem cells (HSCs) were examined three months after the exposure. The results showed that the frequencies and numbers of BM HPCs and HSCs were significantly reduced in 0.1 Gy, 0.25 Gy and 1.0 Gy irradiated mice compared to 0.05 Gy irradiated and non-irradiated mice. Exposure of mice to low dose ^16^O irradiation also significantly reduced the clongenic function of BM HPCs determined by the colony-forming unit assay. The functional defect of irradiated HSCs was detected by cobblestone area-forming cell assay after exposure of mice to 0.1 Gy, 0.25 Gy and 1.0 Gy of ^16^O irradiation, while it was not seen at three months after 0.5 Gy and 1.0 Gy of γ-ray irradiation. These adverse effects of ^16^O irradiation on HSCs coincided with an increased intracellular production of reactive oxygen species (ROS). However, there were comparable levels of cellular apoptosis and DNA damage between irradiated and non-irradiated HPCs and HSCs. These data suggest that exposure to low doses of ^16^O irradiation induces long-term hematopoietic injury, primarily via increased ROS production in HSCs.

## Introduction

Deep space missions are associated with several risk factors, including the exposure to space radiation. Two main sources of space radiation are solar particle events (SPE) and galactic cosmic rays (GCR)[1]. Specifically, high atomic number and energy (HZE) particles from GCR, such as ^56^Fe, ^28^Si, ^16^O, and ^12^C, have higher energy and stronger toxicities to normal tissues than photon and proton radiation[2, 3]. Although exposure to space radiation usually occurs at low doses and dose rates, an extended exposure of the body to space radiation, particularly to HZE radiation, during long-term space missions might result in a dose accumulation of HZE radiation sufficient to cause health hazards[4, 5]. Fornace’s group reported that the value of relative biological effectiveness (RBE) in relation to γ-ray irradiation was 1.25 for ^56^Fe, 1.4 for ^28^Si and 0.99 for ^12^C using a mouse model[2, 6]. However, there is limited knowledge of the RBE of ^16^O ion exposure, especially regarding the long-term health effects, which limits the development of mitigating strategies against space radiation.

A plethora of studies including ours have reported that photon radiation (such as γ-rays) induced both acute and chronic bone marrow (BM) suppression, especially the long-term defect of hematopoietic stem cells (HSCs), resulting from radiation-induced production of reactive oxygen species (ROS), DNA damage, apoptosis, and cellular senescence[7–9]. Previous studies have demonstrated that exposure of mice to high energy ^56^Fe, ^12^C and neutron had detrimental effects on the hematopoietic system, including decreased peripheral blood counts and reduced colony forming ability of HSCs and hematopoietic progenitor cells (HPCs) [2, 10–12]. However, the hematopoietic effects of high energy ^16^O irradiation have been barely studied. In addition, several labs have demonstrated that exposure to protons at low doses significantly decreased the numbers of peripheral blood cells including white blood cells and platelets starting at 4 hours post exposure in porcine and mouse models[13–18]. The abnormal levels of white blood cells in the spleen still existed 110 days after proton exposure[19]. Consistent with these results, we recently reported that low doses of proton total body irradiation (TBI) resulted in long-term detrimental effects on the hematopoietic system[20]. However, we have recently shown that low doses of high HZE TBI in the form of ^16^O ions (^16^O TBI) also caused acute hematopoietic damage, when measured two weeks after TBI in C57BL/6J mice[21]. ^16^O TBI was not only associated with a decrease in peripheral blood cell counts, but also with a functional deficiency in HPCs and HSCs. The acute negative effects on HPC and HSC function coincided with induction of ROS production, DNA damage and apoptosis[21]. However, long-term effects of ^16^O TBI on the hematopoietic system remain unknown and therefore were investigated in the present study.

## Materials and methods

### Animals and irradiation

Male C57BL/6J mice were purchased from the Jackson Laboratory (Bar Harbor, ME) and housed at the University of Arkansas for Medical Sciences (UAMS) until 6 months of age. Mice were administered a standard soy-protein free rodent diet (2020X, Harlan Teklad, Indianapolis, IN) and water *ad libitum* and housed under a constant 12 h light: dark cycle throughout the study. Six-month old mice were shipped to Brookhaven National Laboratories (BNL) in Upton, NY. After a one-week acclimation period, the mice were either sham irradiated or received whole-body ^16^O irradiation (600 MeV/n; 0.05 Gy, 0.1 Gy, 0.25 Gy and 1.0 Gy, 0.25–0.26 Gy/min, n = 5) at the NASA Space Radiation Laboratory (NSRL). For each exposure, animals were individually placed into clear Lucite cubes (3 in x 1½ in x 1½ in) with breathing holes. Sham irradiated mice were placed into the same enclosures for the same amount of time, but were not exposed to radiation. One day after (sham-) irradiation, the mice were returned to UAMS. Mice were administered 2020X chow containing 150 ppm fenbendazole for the first 8 weeks after return, as a routine UAMS quarantine procedure. During the entire experiment, sham-irradiated mice were not housed together with irradiated mice. All procedures in this study were approved by the Institutional Animal Care and Use Committees of UAMS and BNL.

Dosimetry was performed by the NSRL physics group to ensure the quality of exposure. Briefly, the NSRL beamline recorded the charge delivered to a transmission ion chamber placed just in front of the animals. The transmission ion chamber was calibrated against a National Institute of Standards and Technology (NIST)-traceable 1.0 cm^3^ thimble ion chamber (EG&G, Inc.) which was placed at the target position. The doses indicated in the present study were thus the absorbed doses by the animals in the ion chamber at the target surface location on the beamline. The BM of mice is < = 1.0 mm beneath the skin of the animals so the total material between the skin surface and the BM is <2 mm. With particles of multi-centimeter ranges, there is no significant difference in dose as reported at the surface compared to ~1.5 mm depth for the BM. Therefore, the doses of 0.05 Gy, 0.1 Gy, 0.25 Gy and 1.0 Gy ^16^O TBI are the actual doses to the BM in our current study. In addition, we ran a GERM (GCR Event-based Risk Model) code simulation to determine that the LET (Linear Energy Transfer) of 600 MeV/n oxygen ions is 16.45 keV/micron. We applied the following formula to calculate fluence: Dose (cGy) = 1.602x10^-7^ x LET (keV/um) X fluence. The fluence at 0.1 Gy was 3.8E+06 particles/cm^2^. The diameter of hematopoietic stem and progenitor cells is 8.0 microns[22]. Assuming circular cells of 8.0 microns in diameter, a dose of 0.1 Gy oxygen ions (600 MeV/n) will result in an average of 1.91 ion traversals per cell. The average numbers of oxygen ion traversing each cell are 0.96 ion/cell for 0.05 Gy, 1.91 ions/cell for 0.1 Gy, 4.78 ions/cell for 0.25 Gy and 19.1 ions/cell for 1.0 Gy, respectively (Table 1). Based on the poisson distribution of particle traversal, 62% of cells will be traversed by one or more ions and 38% will not be traversed at a dose of 0.05 Gy. However, 99% of cells will be traversed at a dose of 0.25 Gy (Table 1).

**Table 1:** Properties of oxygen ion irradiation.

Doses of ^16^O	Average hits/cell	% of traversed/non-traversed cells
0.05 Gy	0.96	62/38
0.1 Gy	1.91	85/15
0.25 Gy	4.78	99/1
1.0 Gy	19.1	100/0

Total body γ-ray irradiation (γ-TBI) was performed at UAMS. Six-month old mice were put in a plastic box on a rotating platform and exposed to 0.5 Gy and 1.0 Gy of γ-TBI (n = 5) in a Mark I ^137^Ce γ-irradiator (JL Shepherd, Glendale, CA, USA) at a rate of 1.14 Gy/min.

### Tissue and peripheral blood collection

Three months after irradiation, mice were anesthetized and sacrificed with continued exposure to 5% isoflurane for at least 5 min after respiratory arrest. A modified infusion set (27G with shortened tubing) was used to inject a single dose of heparin (30–40 U/kg) into the abdominal vena cava. Without changing the position of the infusion set, a blood sample was drawn, transferred into an EDTA coated tube, and stored on ice until further analysis. Peripheral blood cell counts were determined by a Vet ABC™ Hematological analyzer (SCIL Animal Care Co.). The femora and tibia were collected and immediately processed for cell isolation and analysis as described below.

### Isolation of BM mononuclear cells (BM-MNCs), analysis of the frequencies and numbers of different hematopoietic cell populations by flow cytometry

The femora and tibia were immediately flushed with Hanks' Balanced Salt Solution (HBSS) containing 2% fetal bovine serum (FBS) using a 21-gauge needle and syringe to collect BM cells. BM samples were placed on ice and analyzed within the same day as described below.

For phenotypic analysis, BM cells were incubated with biotin-conjugated anti-CD3e, anti-CD45R/B220, anti-Gr-1, anti-CD11b, and anti-Ter-119 antibodies and with anti-CD16/32 (Fcγ II/III Receptor or FcγR) antibody to block the Fcγ receptors. Cells were then labeled with streptavidin-FITC, anti-Sca-1-PE-Cy7, anti-c-Kit-APC-Cy7, anti-CD150-APC and anti-CD48-Pacific blue for HPCs (Lin^-^Sca1^-^c-kit^+^ cells), LSK cells (Lin^-^Sca1^+^c-kit^+^ cells), and HSCs (Lin^-^Sca1^+^c-kit^+^CD150^+^CD48^-^ cells). Dead cells were excluded by gating out the cells stained positive with propidium iodide (PI). The frequencies of HPCs and HSCs were analyzed with an Aria II cell sorter and presented as percentages of total living BM cells. The numbers of different hematopoietic cell populations in each mouse were calculated by multiplying the total numbers of BM cells harvested from the two hind legs of each mouse with the frequencies of each population in the cells. For each sample, approximately 8 x 10^5^ to 1 x 10^6^ BM cells were acquired and the data were analyzed using BD FACSDiva 6.0 (BD Biosciences) and FlowJo (FlowJo, Ashland, OR) software.

For the isolation of Lineage negative cells (Lin^-^ cells), BM-MNCs were isolated by Histopaque 1083 separation solution (Sigma, St. Louis, MO). BM-MNCs were incubated with purified rat antibodies specific for murine CD3e, Mac-1, CD45R/B220, Ter-119, and Gr-1. The labeled mature lymphoid and myeloid cells were depleted by incubating with goat anti-rat IgG paramagnetic beads (Life Technologies, Grand Island, NY) at a bead:cell ratio of approximately 4:1. Cells binding the paramagnetic beads were removed with a magnetic field. Lin^-^ cells were washed twice with 2% FBS/HBSS and resuspended in complete medium (RPMI1640 medium supplemented with 10% FBS, 2 mM L-glutamine, 10 μM HEPES buffer, and 100 U/mL penicillin and streptomycin) at 1x10^7^ cells/mL. Lin^-^ cells were subsequently used to analyze cellular apoptosis, ROS production, DNA damage and cell cycle in HPCs, LSK cells and HSCs as shown below. All flow antibodies were purchased from eBioscience (San Jose, CA).

### Colony-forming unit (CFU) assay

The CFU assay was performed by culturing BM-MNCs in MethoCult GFM3434 methylcellulose medium (Stem Cell Technologies, Vancouver, BC). 2×10^4^ BM-MNCs were seeded into each well of 12-well plates. The numbers of CFU-granulocyte macrophage (CFU-GM) and burst-forming unit-erythroid (BFU-E) were scored under microscopy on day 7 and those of CFU-granulocyte, -erythrocyte, -monocyte, and -megakaryocyte (GEMM) were counted on day 12 after cells were seeded.

### Cobblestone area-forming cell (CAFC) assay

1×10^3^ FBMD-1 stromal cells were seeded in each well of flat-bottomed 96-well plates (Falcon, Lincoln Park, NJ). One week later, BM-MNCs from irradiated and non-irradiated mice were suspended in CAFC medium (Iscove’s MDM supplemented with 20% horse serum, 10^−5^ M hydrocortisone, 10^−5^ M 2-mercaptoethanol, 100 units/ml penicillin, and 100 μg/ml streptomycin). Cells were then overlaid on the stromal layers in six dilutions and 3-fold apart. Twenty wells were plated for each dilution to allow limiting dilution analysis of the precursor cells forming hematopoietic clones under the stromal layer. Cells were fed weekly by changing one-half of the medium. Wells were scored positive if at least one phase-dark hematopoietic clone (containing 5 or more cells) was seen. The frequencies of CAFC were determined at week 5 to determine the clonogenic function of HSCs. The frequency of CAFC was then calculated by using Poisson statistics.

### Apoptosis assay

Lin^-^ cells were incubated with anti-CD16/32 at 4°C for 15 min to block the Fc-γ receptors and then stained with antibodies against various cell surface markers in the dark. After Annexin V staining with a kit from BD Pharmingen (San Diego, CA) according to the manufacturer’s instructions, apoptotic cells in different hematopoietic cell populations were analyzed with an Aria II cell sorter.

### Analysis of the levels of intracellular ROS

After staining with the appropriate cell surface marker antibodies, Lin^-^ cells (1 x 10^7^/mL) were suspended in PBS supplemented with 5 mM glucose, 1 mM CaCl_2_, 0.5 mM MgSO_4_, and 5 mg/ml BSA and then incubated with 10 μM 2',7'-dichlorofluorescein diacetate (DCFDA) (Life Technologies, Grand Island, NY) for 30 minutes at 37°C. The levels of ROS in HPCs and HSCs were analyzed by measuring the mean fluorescence intensity (MFI) of 2',7'-dichlorofluorescein (DCF) with an Aria II cell sorter. For each sample, a minimum of 200,000 lineage negative cells was acquired and the data were analyzed as previously described [23].

### DNA damage analysis

Lin^-^ cells were first stained with antibodies against various cell-surface markers and fixed and permeabilized using the Fixation/Permeabilization Solution from BD Biosciences (San Diego, CA) followed by 0.2% Triton-X-100 incubation for 10 min. Cells were then stained with Alexa Fluor 488 conjugated anti-phospho-Histone 2AX (Ser139) (γH2AX, Cell Signaling Technology, MA) antibody for 1.5 hours at 4°C and analyzed by flow cytometry. The levels of DNA double strand break damage were expressed by the mean fluorescence intensity (MFI) of γH2AX with an Aria II cell sorter.

### Cell cycle analysis

Lin^-^ cells were first stained with antibodies against various cell-surface markers and fixed and permeabilized using the Fixation/Permeabilization Solution (BD Biosciences, CA). Subsequently, they were stained with anti-Ki67-FITC antibody (BD Biosciences, CA) and 7-aminoactinomycin (7-AAD, Sigma, St. Louis, MO) and then analyzed by flow cytometry.

### Quantitative reverse transcription polymerase chain reaction (qRT-PCR)

HPCs and HSCs for 1.0 Gy ^16^O TBI and non-irradiated mice were sorted out. Total RNA of HPCs and HSCs was isolated using the Qiagen RNeasy Mini Kit (Valencia, CA) according to the manufacturers’ instructions. RNA yield and quality were determined by measuring absorbencies at 260 nm and 280 nm, respectively. First-strand cDNA was synthesized in a final volume of 20 μl using the Superscript III First-Strand Synthesis System (Invitrogen, Carlsbad, CA). qRT-PCR analyses were performed using a SYBR Green mix on an ABI StepOne Plus Real-Time PCR System (Applied Biosystems, Foster City, CA). *Hypoxanthinephophoribosyltransferase (HPRT)* transcripts were used as a housekeeping internal reference for mRNA. The expression of Catalase, GPX1 (Glutathione Peroxidase 1), GPX2, SOD1 (Superoxide Dismutase 1), SOD2 and SOD3 was calculated by the comparative C_T_ method. The sequences for all the primers used in the qRT-PCR assays are available upon request.

### Statistical analysis

All data are presented as mean ± standard deviation of three to five independent biological samples per radiation dose. The differences between sham-irradiated and irradiated groups were examined by one-way ANOVA, followed by Tukey-Kramer test for individual comparisons. Differences were considered significant at *p* < .05. Statistical analysis was performed using GraphPad Prism (GraphPad Software Inc. LaJolla, CA).

## Results

### ^16^O TBI induced long-term BM suppression

**Fig 1:**
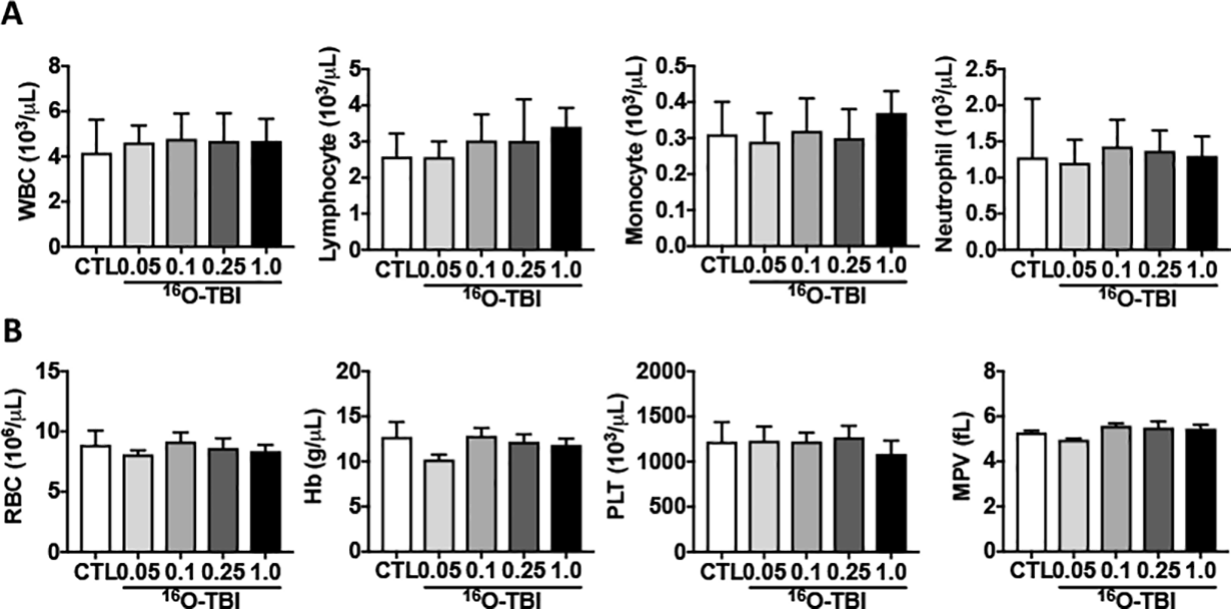
Peripheral blood cell counts were comparable between non-irradiated and irradiated mice at three months after ^16^O exposure. C57BL/6J mice were exposed to 0.05 Gy, 0.1 Gy, 0.25 Gy and 1.0 Gy doses of ^16^O irradiation or were sham irradiated as a control (CTL). The cell counts in peripheral blood were determined three months after radiation exposure. (A-C) The numbers of WBC, lymphocytes, monocytes, neutrophils, RBC, Hb, platelet (PLT) and mean platelet volume (MPV) in irradiated mice are presented as means ±SD (n = 5), and comparable to those in non-irradiated mice.

**Fig 2:**
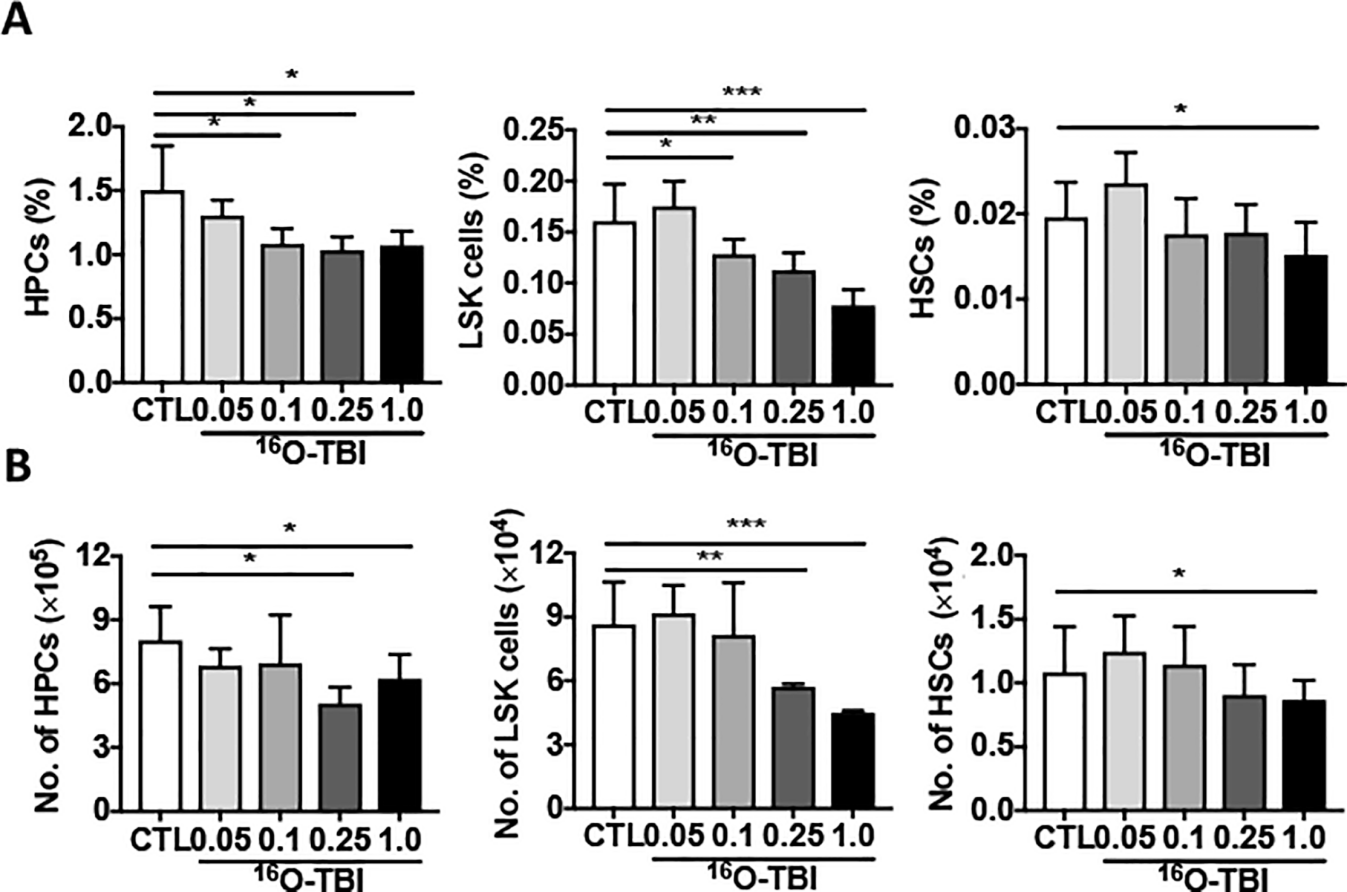
^16^O TBI caused reductions in percentages and numbers of HPCs, LSK cells and HSCs at three months after exposure. HPCs (Lin^-^Sca1^-^c-kit^-^ cells), LSK cells (Lin^-^Sca1^+^c-kit^+^cells) and HSCs (Lin^-^Sca1^+^c-kit^+^CD150^+^CD48^-^ cells) in BM were measured three months after 0.05 Gy, 0.1 Gy, 0.25 Gy, and 1.0 Gy ^16^O TBI. The frequencies (panel A) and numbers (panel B) of HPCs, LSK cells and HSCs from total bone marrow cells in each mouse are presented as means ±SD (n = 5). The statistical significance for differences between the control group (CTL) and each of the irradiated groups is indicated by asterisks. *p<0.05, **p<0.01, ***p<0.001 as determined by one-way ANOVA, followed by Tukey-Kramer test for individual comparisons.

We have previously shown that 1.0 Gy of ^16^O TBI significantly decreased peripheral blood cell counts, particularly those of white blood cells (WBC) and platelets (PLT), in mice two weeks after TBI[21]. To assess the chronic effects of ^16^O TBI on peripheral blood cells, we collected peripheral blood at three months after 0.05 Gy, 0.1 Gy, 0.25 Gy and 1.0 Gy of ^16^O TBI. As shown in Fig 1, the numbers of all different types of peripheral blood cells including WBC, PLT and red blood cells had reached normal levels, regardless of radiation doses. Peripheral blood cells were also recovered at three months after 0.5 Gy and 1.0 Gy of γ-ray irradiation (S1 Fig). However, the data from peripheral blood cell counts could not infer potential changes in BM HPCs and HSCs after TBI as shown in our previous studies [21]. Therefore, the frequencies and numbers of different BM hematopoietic cell populations were analyzed by flow cytometry (S2 Fig). As shown in Fig 2A, compared to 0.05 Gy ^16^O TBI and non-irradiated controls, the frequency of HPCs (Lin^-^Sca1^-^c-kit^+^ cells) was significantly reduced in all other three doses of ^16^O-irradiated mice (*p*<0.05). Moreover, the frequency of LSK cells (Lin^-^Sca1^+^c-kit^+^cells) was decreased in 0.1 Gy-, 0.25 Gy- and 1.0 Gy-irradiated mice compared to that in 0.05 Gy irradiated and non-irradiated mice (*p*<0.05-*p<*0.001). While the frequency of HSCs (Lin^-^Sca1^+^c-kit^+^CD150^+^CD48^-^cells) in 1.0 Gy irradiated mice was decreased compared to that in 0.05 Gy-, 0.1 Gy-, 0.25 Gy- and non-irradiated mice(*p*<0.05), the frequencies of HSCs in 0.05 Gy-, 0.1 Gy-, 0.25 Gy-irradiated mice were comparable to those in non-irradiated mice. The total numbers of HPCs, LSK cells and HSCs showed trends similar to those of the frequencies of each of these cell populations (Fig 2B, *p*<0.05–0.001). There were increasing trends in both frequencies and numbers of HSCs from 0.5 Gy and 1.0 Gy γ-irradiated mice without reaching statistical significance (S3A and S3B Fig). This might be due to the variations in HSCs from non-irradiated mice. The frequencies and numbers of HPCs and LSK cells from γ-irradiated mice were comparable to those in non-irradiated controls at three months after 0.5 Gy and 1.0 Gy γ-ray exposure (S3A and S3B Fig).

**Fig 3:**
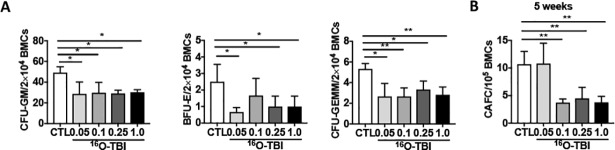
^16^O TBI caused the reduction of the clonogenic function in HPCs and HSCs. (A) BM-MNCs were isolated from irradiated and non-irradiated (CTL) mice three months after ^16^O TBI and a CFU assay was performed. Results are presented as mean CFUs per 1x10^5^ BM-MNCs (n = 5). (B) Total BM cells (BMCs) were analyzed by CAFC assays, and the numbers of five-week CAFCs are expressed as means ± SD (n = 3 mice per group) per 1x10^5^ BMCs. The statistical significance for differences between the control group and each of the irradiated groups is indicated by asterisks. *p<0.05, **p<0.01 as determined by one-way ANOVA, followed by Tukey-Kramer test for individual comparisons.

To further evaluate the effect of ^16^O TBI on BM HPCs and HSCs, a colony forming assay was used to examine their clonogenic function. The results showed that the frequencies of BFU-E, CFU-GMs, and CFU-GEMMs in BM cells were significantly reduced, regardless of radiation doses (Fig 3A, *p*<0.05–0.01), indicating that the clonogenic function of HPCs was dramatically impaired in all irradiated mice, while the decreased colony forming abilities of HPCs and HSCs were not seen three months after 0.5 Gy and 1.0 Gy γ-TBI (S3C Fig). In addition, we used another standard *in vitro* HSC functional assay named the cobblestone area forming assay (CAFC) to assess the function of HSCs. The data showed that the frequencies of 5-week CAFCs were significantly lower in BM cells from 0.1 Gy, 0.25 Gy and 1.0 Gy ^16^O-irradiated mice than those from 0.05 Gy ^16^O-irradiated mice and non-irradiated controls (Fig 3B, p<0.01). However, there were comparable numbers of CAFCs formed in 0.1 Gy, 0.25 Gy and 1.0 Gy ^16^O-irradiated mice. The frequencies of 5-week CAFCs in 0.05 Gy ^16^O-irradiated mice were equal to those in non-irradiated mice. These data indicate that exposure of mice to ^16^O TBI, especially higher than 0.05 Gy ^16^O TBI, causes long-term damage not only to HPCs but also to HSCs.

### ^16^O TBI induced oxidative stress in HSCs

**Fig 4:**
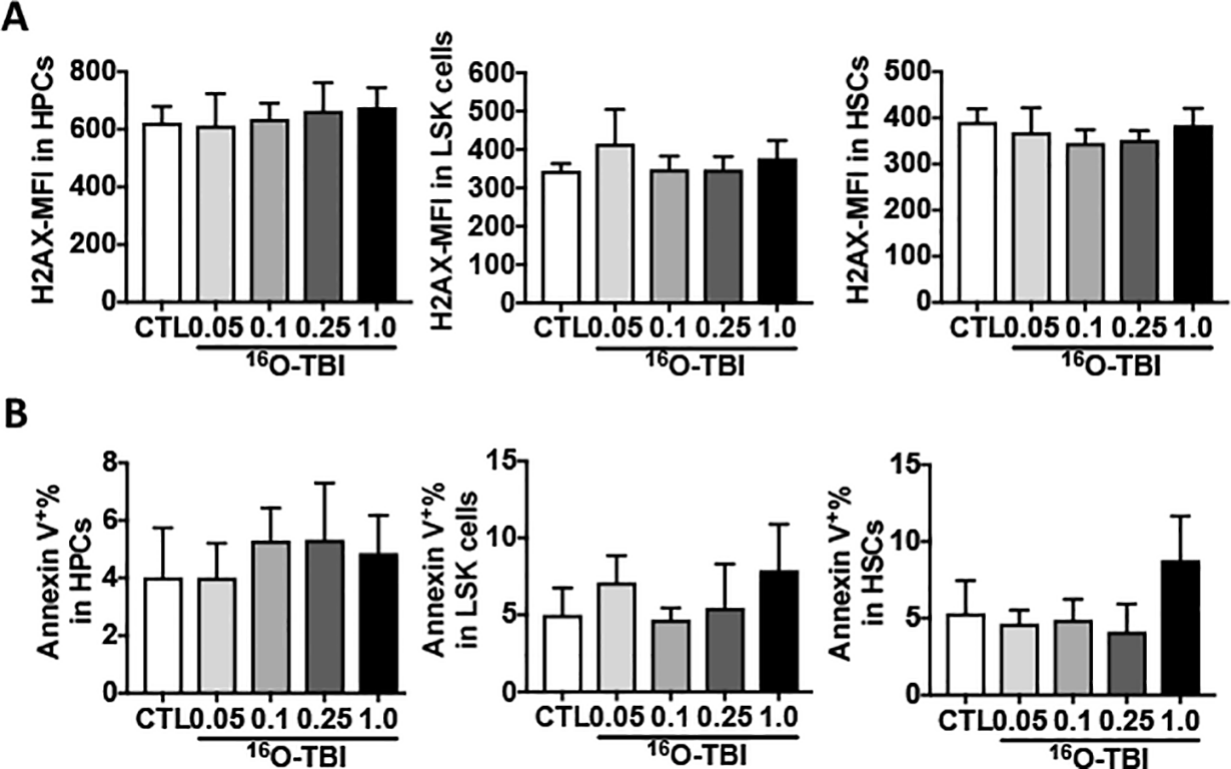
No changes were detected in DNA damage and apoptosis in HPCs, LSK cells and HSCs at three months after ^16^O TBI. (A) Lin^-^ cells were stained with an anti-γH2AX antibody and analyzed by flow cytometry. Data are presented as mean fluorescence intensity (MFI). (B) Isolated Lin^-^ cells were stained with Annexin V to determine cellular apoptosis. Percentages of Annexin V positive cells are presented as means ± SD (n = 5).

Exposure to ionizing radiation can cause oxidative stress, DNA damage and apoptosis or senescence [23]. To test these possibilities, we first used γH2AX immunostaining to assess DNA double strand breaks in HPCs and HSCs after ^16^O TBI. The immune fluorescence intensities (MFI) of γH2AX in HPCs, LSK cells and HSCs were comparable between non-irradiated and irradiated cells three months after ^16^O TBI (Fig 4A). We then tested apoptosis in different populations using Annexin V staining. As shown in Fig 4B, there were no differences in the percentage of apoptotic cells between irradiated and non-irradiated HPCs. Compared to 0.05 Gy-, 0.1 Gy-, 0.25 Gy- and non-irradiated groups, LSK cells and HSCs exposed to 1.0 Gy showed a trend of 5–10% increase in apoptosis without reaching a statistical significance.

**Fig 5:**
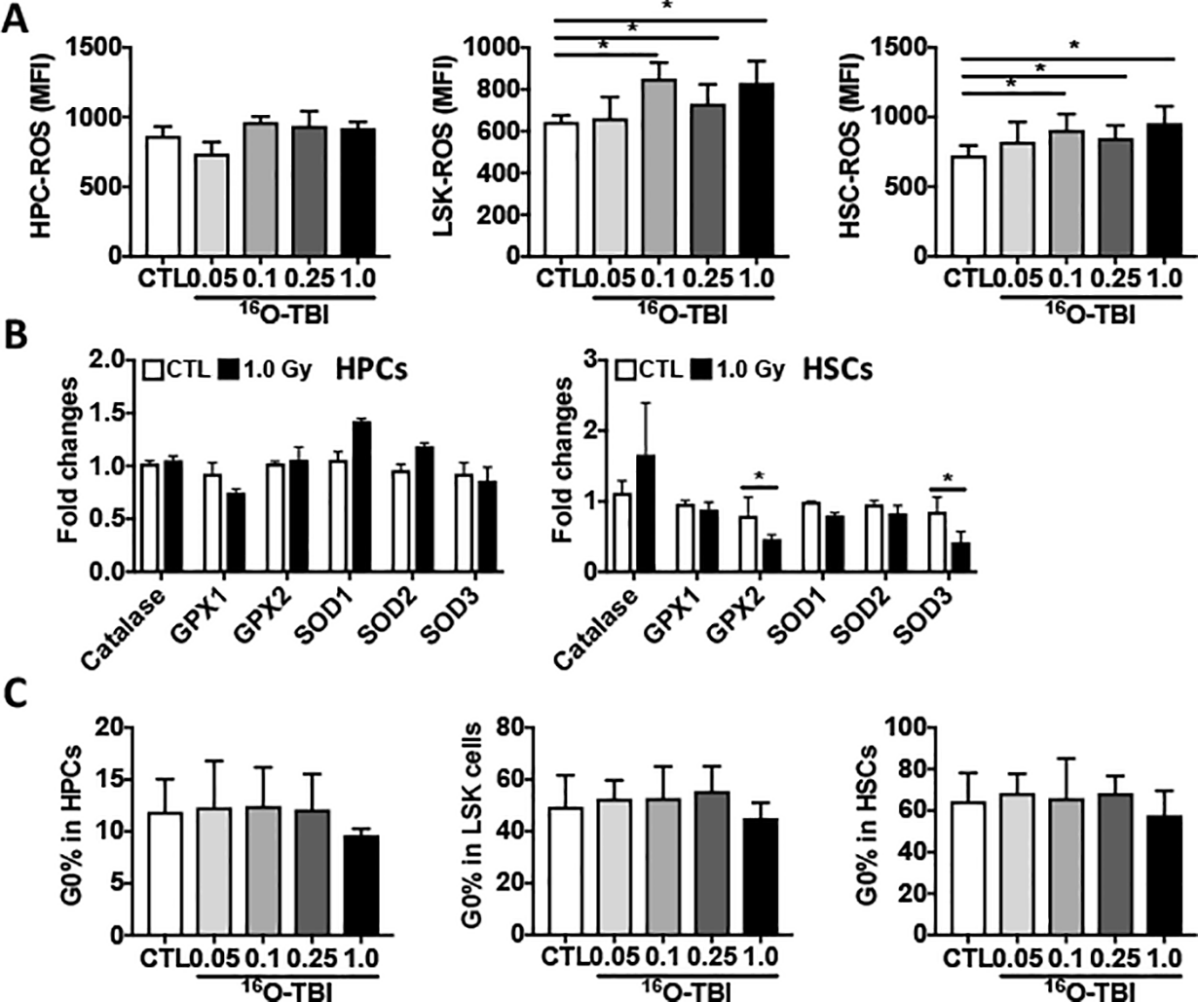
^16^O TBI caused an increase in ROS production in LSK cells and HSCs three months after the exposure. (A) Lin^-^ cells were used to measured ROS production by staining with DCFDA and analyzed by flow cytometry. The DCF mean fluorescence intensity (MFI) in BM HPCs and HSCs are presented as means ± SD (n = 5). (B) Fold changes in relative gene expression for several antioxidant genes in sorted HPCs (left panel) and HSCs (right panel) from 1.0 Gy of ^16^O TBI and non-irradiated mice. (C) Lin^-^ cells were isolated and cell cycling was measured by cytometry using Ki-67 and 7-AAD double staining in HPCs and HSCs from control (CTL) and irradiated mice. The statistical significance for differences between the control group and each of the irradiated groups is indicated by asterisks. *p<0.05, **p<0.01, ***p<0.001 as determined by one-way ANOVA, followed by Tukey-Kramer test for individual comparisons.

In addition to DNA damage and apoptosis, ionizing radiation can also affect cellular function through induction of oxidative stress and alterations in cell cycling [23]. To determine whether induction of oxidative stress may mediate the effects of ^16^O TBI on BM HPCs and HSCs, the intracellular production of ROS in HPCs and HSCs was assessed. As shown in S4 Fig, the distribution of ROS in non-irradiated and 1.0 Gy ^16^O-irradiated HPCs was similar, while the distribution of ROS in 1.0 Gy ^16^O-irradiated LSK cells and HSCs was different from that in non-irradiated LSK cells and HSCs. Consistently, ROS production in irradiated HPCs was comparable to that in cells from non-irradiated mice (Fig 5A). There were significantly higher levels of ROS production (up to 30% increase of normal levels) in LSK cells and HSCs from mice exposed to 0.1 Gy, 0.25 Gy and 1.0 Gy of ^16^O TBI compared to that in 0.05 Gy ^16^O TBI and non-irradiated cells (Fig 5A, *p*<0.05). However, the levels of ROS production in LSK cells and HSCs from mice exposed to different doses of ^16^O were not significantly different. To determine whether the increase in ROS production was attributable to a decreased expression of antioxidant enzymes, we sorted out HPCs and HSCs from 1.0 Gy ^16^O irradiated and non-irradiated mice to perform gene expression analysis. As shown in Fig 5B, there were significant lower levels of GPX2 and SOD3 expression in irradiated HSCs compared to those in non-irradiated HSCs (*p*<0.05). No difference was observed in the expression of catalase, GPX1, SOD1 and SOD2 between irradiated and non-irradiated HSCs. There were comparable expression levels of these antioxidant genes in irradiated and non-irradiated HPCs (Fig 5B). We also utilized Ki-67 and 7-AAD double immunostaining to measure the cell cycle status of HPCs and HSCs after ^16^O exposure. Comparable percentages of cells in G_0_ phase were detected in HPCs, LSK cells and HSCs from non-irradiated and irradiated mice (Fig 5B), suggesting that the status of the cell cycle in BM HPCs and HSCs was recovered at 3 months after ^16^O irradiation. However, no increased DNA damage, apoptosis, ROS and cell cycle in irradiated HPCs and HSCs were detected three months after 0.5 Gy and 1.0 Gy of γ-ray exposure (S5A and S5B Fig, data not shown).

## Discussion

Based on measurements in the Mars Science Laboratory in 2011–2012, the exposure to GCR inside the space craft was estimated to be 481 ± 80 μGy per day or 176 ± 29 mGy per year [24]. To straddle this estimated annual dose, we selected doses from 0.05 Gy to 0.25 Gy in the range of expected doses for extended lunar and deep space missions. For a 600- to 900- day Mars mission, the contributions from GCR would be 0.33 to 0.49 Gy (neglecting protons from solar particle events which could bring total exposures close to 1.0 Gy). Therefore, 1.0 Gy high dose of ^16^O irradiation allows us to span the range of expected values and enables us to detect curvature in our dose responses. Because some doses used in the present study are higher than those exposed by astronauts during space flights, cautions should be taken when the findings from our mouse study are extrapolated to humans. During long-term space missions, astronauts will be exposed to space irradiation in a chronic or fractionated manner. To better mimic HZE exposure in deep space, chronic or fractionated irradiation should be used in the actual studies. However, these are not currently achievable because of radiation source limiting access. In the present report, the acute dose rate (0.25–0.26 Gy/min) was used with a limitation of current study, while these practical limitations may not invalidate the value of the study.

In our previous study, we evaluated the acute effects of ^16^O TBI on the hematopoietic system two weeks after irradiation in a mouse model and showed that ^16^O TBI acutely decreased various peripheral blood cell counts and the frequency and number of BM HPCs and HSCs[21]. The present study extended previous investigations using four different doses of ^16^O TBI to determine long-term effects of ^16^O TBI on the hematopoietic system. We are the first to demonstrate that exposure to low doses of ^16^O TBI induces long-term damage to BM HPCs and HSCs in mice, but without a significant effect on the numbers of WBC, RBC and PLT. These long-term effects of ^16^O irradiation on peripheral blood cells are similar to the effects of other types of ionizing radiation including currently used 0.5 Gy and 1.0 Gy of γ-TBI[25, 26]. For instance, the restoration of peripheral blood cell counts to normal levels could be seen two months after γ-ray exposure[27]. However, through analysis of BM HPCs and HSCs, we show that the seemingly complete recovery of peripheral blood cell counts may obscure the discovery of long-term BM suppression induced by radiation[25], which may cause this important pathology to be overlooked.

Studies from other labs and ours have demonstrated that sublethal doses of γ-rays (such as 6.5 Gy γ-TBI) and low doses of proton radiation resulted in long-term residual damage in the hematopoietic system[23, 26, 28, 29], as evidenced by a reduction in HSC reserves and a defect in HSC function. Consistent with these previous results, the current study showed that low doses of ^16^O TBI, especially higher than 0.05 Gy of ^16^O TBI, induced a significant decrease in the numbers and function of LSK cells and HSCs in BM three months after exposure. However, it was not seen at three months after 0.5 Gy and 1.0 Gy of γ-TBI. On the other hand, while the numbers of HPCs in irradiated mice reached normal levels two weeks after γ-ray and proton exposure, our data indicate that acute exposure to low doses of ^16^O TBI causes significant reductions in numbers of HPCs at 2 weeks and 3 months after the exposure [21]. These results suggest that ^16^O irradiated HPCs experience a slower recovery than proton- and photon-irradiated HPCs. This may be related to the higher RBE [30] and potential bystander effects of ^16^O irradiation. According to the calculation of the average numbers of oxygen ion traversing cells, each cell will experience 1.91 ions for 0.1 Gy exposure and 4.78 ions for 0.25 Gy exposure. After applying a poisson distribution (Table 1), we estimate that at a dose of 0.1 Gy 85% of cells will be traversed by one or more ions and 15% will not be traversed. Therefore, non-irradiated cells (15%) might produce irradiated effects because of the signals from irradiated cells (85%). On the other hand, a dose of 0.25 Gy would result in 99% of cells being traversed. Due to the poisson distribution of particle traversal, traversed cells will experience different doses at low doses of exposure, which might cause heterogenous responses in irradiated hematopoietic cells.

The sensitivity of HPCs and HSCs to ionizing radiation is usually examined by evaluating both cell numbers and their function. We have previously shown that HPCs, but not HSCs, showed high levels of apoptosis two weeks after 1.0 Gy of ^16^O TBI, together with a decrease in the HPC colony forming abilities as indicated by lower numbers of BFU-E, CFU-GM and CFU-GEMM compared to those in non-irradiated HPCs[21]. The current study shows that HPC colony forming abilities remained much lower compared to normal controls three months after ^16^O TBI. Moreover, the decreased colony forming abilities of irradiated HPCs were radiation dose-independent in the dose-range used here, which suggests “hit and damage”. However, we don’t know the underlying mechanisms of the decreased HPC function after low doses of ^16^O TBI, since apoptosis, ROS production and DNA damage in irradiated HPCs were comparable with non-irradiated controls.

Our *in vitro* CAFC assay demonstrated that ^16^O TBI reduced the long-term colony forming capacities of HSCs after 0.1 Gy, 0.25 Gy and 1.0 Gy exposure, again indicating “hit and damage”. However, there were comparable numbers of 5-week CAFCs between 0.05 Gy irradiated mice and non-irradiated controls, suggesting that HSC function was not reduced after exposure to 0.05 Gy ^16^O TBI. These long-term effects of ^16^O TBI on HSCs are similar to those of other types of ionizing radiation[20, 31, 32]. For example, 1.0 Gy of proton radiation decreased the CAFC forming ability in HSCs at 22 weeks in a mouse model[20]. A dose of 6.5 Gy of γ-rays reduced HSC colony forming ability 2 months after the exposure[7]. These findings indicate that long-term HSC suppression occurs upon low doses of ionizing radiation, including ^16^O, but ^16^O is more potent to induce the long-term HSC suppression than other types of ionizing radiation. Although the numbers and function of HSCs in BM were decreased upon 1.0 Gy of ^16^O TBI, the counts of peripheral blood cells were back to normal levels at 3 months after exposure. Proliferation of progenitors (such as myeloid, lymphoid and erythroid progenitors) might compensate for the decrement of irradiated HSCs, which would cause a transient decrease of these progenitors at a certain time point after irradiation. Notably, the decrease of HSC functions induced by ^16^O irradiation appeared in all irradiated mice in a dose-independent manner. These unusual dose-response curves have also been seen in the studies of ^28^Si irradiation, such as effects of ^28^Si irradiation on synaptic plasticity and contextual fear memory [33, 34].

Previously, we demonstrated that the acute adverse effects of ^16^O TBI on HSCs could be ascribed to induction of apoptosis, DNA damage, ROS production and enhanced cell cycling[21]. In this study, we extended these studies by measuring these parameters in HSCs three months after ^16^O TBI. While the levels of apoptosis, DNA damage and cell cycling were comparable to those in non-irradiated HSCs, there was a consistent increase in ROS production in irradiated HSCs from ^16^O TBI mice. Consistently, the expression of the antioxidant genes GPX2 and SOD3 was significantly decreased in 1.0 Gy ^16^O irradiated HSCs compared to non-irradiated HSCs. The long-term negative effects of ROS accumulation in HSCs have been studied extensively using proton radiation and γ-rays[20, 31, 35, 36]. For example, we have previously shown that both protons and γ-rays induced an increase in ROS in HSCs, together with a decrease in *in vitro* colony forming ability and *in vivo* engraftment capacity. Our recent data showed that γ-Tocotrienol (γ-GT3) treatment significantly increased the capacities of hematopoietic stem and progenitors to forming colonies at three months after 0.25 Gy of ^16^O TBI. This is because ^16^O TBI-induced oxidative stress in irradiated HSCs was significantly decreased after γ-GT3 treatment (unpublished data). These data suggest that oxidative stress may be a major detrimental player in abnormal HSC function in response to ionizing radiation, including ^16^O. In addition, several studies demonstrated that low doses of space ion radiation (such as proton, ^56^Fe and ^28^Si) induced chromosomal aberrations in human lymphocytes and mouse BM cells [37–40], which may predispose irradiated individuals to leukemia. Thus, it is necessary to further assess whether low doses of ^16^O radiation induce chromosomal aberrations in near future, which might be a potential risk factor of ^16^O irradiation-induced hematopoietic malignancies.

Our present study demonstrates long-term effects of ^16^O TBI on HSCs. To minimize the health effects of deep space travel, decreasing oxidative stress might be a good approach to mitigate the adverse effects of HZE particle exposure on the hematopoietic system.

## Supporting information

S1 Fig Peripheral blood cell counts were comparable between non-irradiated and irradiated mice at three months after γ-ray exposure. C57BL/6J mice were exposed to 0.5 Gy and 1.0 Gy doses of γ–ray irradiation (γ-TBI) or were sham irradiated as a control (CTL). The cell counts in peripheral blood were determined three months after radiation exposure. (A-C) The numbers of WBC, lymphocytes, monocytes, neutrophils, RBC, Hb, platelet (PLT) and mean platelet volume (MPV) in irradiated mice are presented as means ±SD (n = 5), and comparable to those in non-irradiated mice.
(TIF)

S2 Fig Flow cytometric gating strategies for various populations of hematopoietic cells in bone marrow. Representative gating strategy of flow cytometric analysis for HPCs (Lin^-^Sca1^-^c-kit^-^ cells), LSK cells (Lin-Sca1^+^c-kit^+^cells) and HSCs (Lin^-^Sca1^+^c-kit^+^CD150^+^CD48^-^ cells) in bone marrow is shown from 1.0 Gy of ^16^O-TBI and sham-irradiation (CTL).
(TIF)

S3 Fig Percentages and numbers of HPCs, LSK cells and HSCs were recovered at three months after γ-ray exposure. (A and B) HPCs, LSK cells and HSCs in BM were measured three months after 0.5 Gy and 1.0 Gy γ-TBI. The frequencies (panel A) and numbers (panel B) of HPCs, LSK cells and HSCs from total bone marrow cells in each mouse are presented as means ±SD (n = 5). (C) BM-MNCs were isolated from irradiated and non-irradiated (CTL) mice three months after γ-TBI and a CFU assay was performed. Results are presented as mean CFUs per 2x10^4^ BM-MNCs (n = 5). The statistical significance for differences between the control group and each of the irradiated groups was determined by one-way ANOVA, followed by Tukey-Kramer test for individual comparisons.
(TIF)

S4 Fig Representative distribution of ROS production in HPCs, LSK cells and HSCs from non-irradiated and 1.0 Gy ^16^O-irradiated mice. Lin^-^ cells were stained with the probe DCFDA and various surface markers, and analyzed by flow cytometry. The distribution and mean fluorescence intensity (MFI) of ROS in non-irradiated and irradiated HPCs, LSK cells and HSCs were presented.
(TIF)

S5 Fig No changes were detected in ROS production and apoptosis in HPCs, LSK cells and HSCs at three months after γ-TBI. (A) Lin^-^ cells were used to measured ROS production by staining with DCFDA and analyzed by flow cytometry three months after 0.5 Gy and 1.0 Gy γ-TBI. The DCF mean fluorescence intensity (MFI) in BM HPCs, LSK cells and HSCs are presented as means ± SD (n = 5). (B) Isolated Lin^-^ cells were stained with Annexin V to determine cellular apoptosis. Percentages of Annexin V positive cells are presented as means ± SD (n = 5).
(TIF)
