# Supplementary figures and images for: Low doses of oxygen ion irradiation cause long-term damage to bone marrow hematopoietic progenitor and stem cells in mice
Source: PLoS One. 2017 Dec 12;12(12):e0189466. doi: 10.1371/journal.pone.0189466 (PMC5726652; doi:10.1371/journal.pone.0189466)

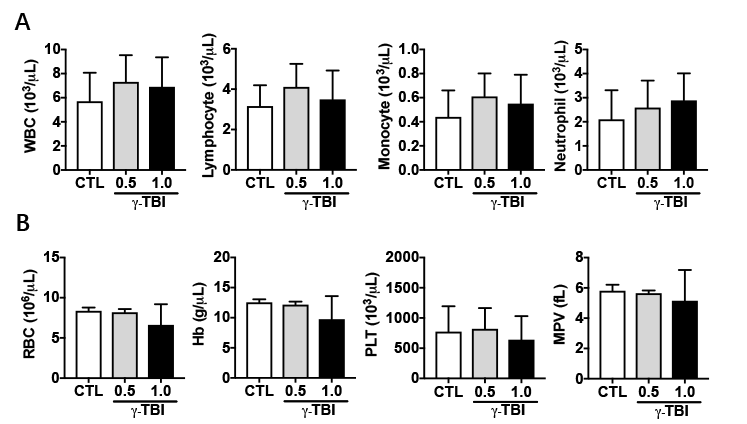

Supplement: S1 Fig — C57BL/6J mice were exposed to 0.5 Gy and 1.0 Gy doses of γ–ray irradiation (γ-TBI) or were sham irradiated as a control (CTL). The cell counts in peripheral blood were determined three months after radiation exposure. (A-C) The numbers of WBC, lymphocytes, monocytes, neutrophils, RBC, Hb, platelet (PLT) and mean platelet volume (MPV) in irradiated mice are presented as means ±SD (n = 5), and comparable to those in non-irradiated mice. (TIF) [file pone.0189466.s001.tif]

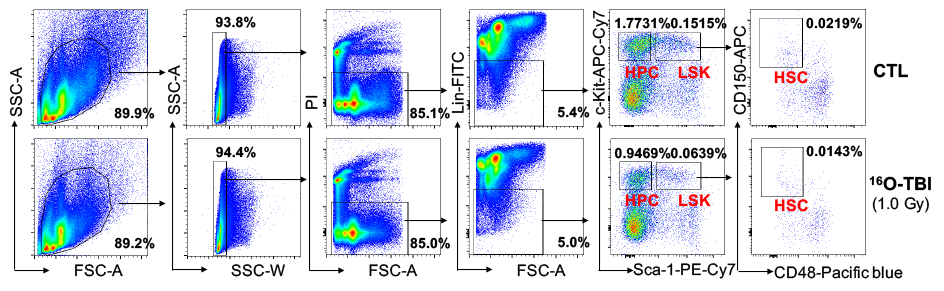

Supplement: S2 Fig — Representative gating strategy of flow cytometric analysis for HPCs (Lin-Sca1-c-kit- cells), LSK cells (Lin-Sca1+c-kit+cells) and HSCs (Lin-Sca1+c-kit+CD150+CD48- cells) in bone marrow is shown from 1.0 Gy of 16O-TBI and sham-irradiation (CTL). (TIF) [file pone.0189466.s002.tif]

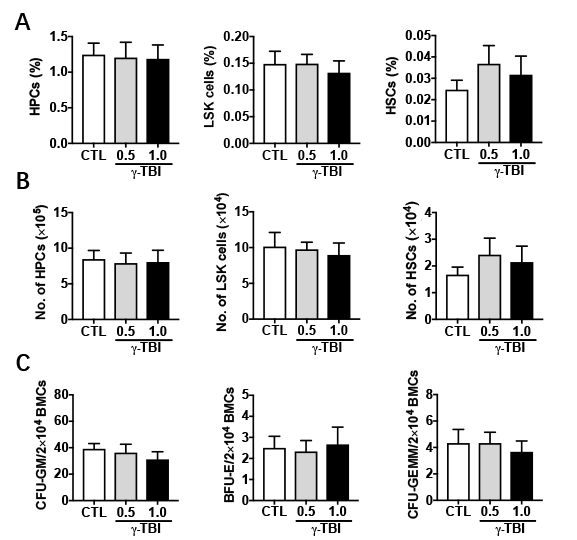

Supplement: S3 Fig — (A and B) HPCs, LSK cells and HSCs in BM were measured three months after 0.5 Gy and 1.0 Gy γ-TBI. The frequencies (panel A) and numbers (panel B) of HPCs, LSK cells and HSCs from total bone marrow cells in each mouse are presented as means ±SD (n = 5). (C) BM-MNCs were isolated from irradiated and non-irradiated (CTL) mice three months after γ-TBI and a CFU assay was performed. Results are presented as mean CFUs per 2x10^4^ BM-MNCs (n = 5). The statistical significance for differences between the control group and each of the irradiated groups was determined by one-way ANOVA, followed by Tukey-Kramer test for individual comparisons. (TIF) [file pone.0189466.s003.tif]

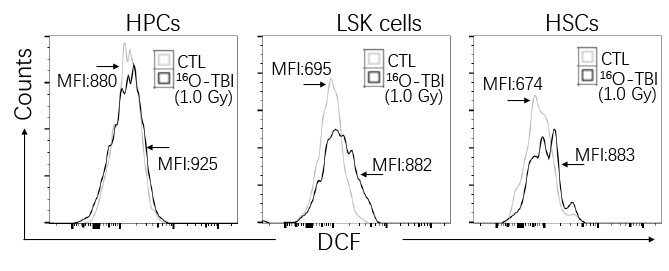

Supplement: S4 Fig — Lin- cells were stained with the probe DCFDA and various surface markers, and analyzed by flow cytometry. The distribution and mean fluorescence intensity (MFI) of ROS in non-irradiated and irradiated HPCs, LSK cells and HSCs were presented. (TIF) [file pone.0189466.s004.tif]

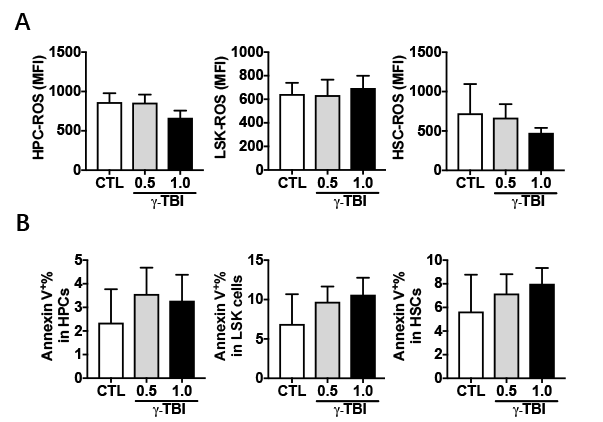

Supplement: S5 Fig — (A) Lin- cells were used to measured ROS production by staining with DCFDA and analyzed by flow cytometry three months after 0.5 Gy and 1.0 Gy γ-TBI. The DCF mean fluorescence intensity (MFI) in BM HPCs, LSK cells and HSCs are presented as means ± SD (n = 5). (B) Isolated Lin- cells were stained with Annexin V to determine cellular apoptosis. Percentages of Annexin V positive cells are presented as means ± SD (n = 5). (TIF) [file pone.0189466.s005.tif]
